# Plasma surfactant protein-D as a diagnostic biomarker for acute respiratory distress syndrome: validation in US and Korean cohorts

**DOI:** 10.1186/s12890-017-0532-1

**Published:** 2017-12-15

**Authors:** Jinkyeong Park, Maria Pabon, Augustine M. K. Choi, Ilias I. Siempos, Laura E. Fredenburgh, Rebecca M. Baron, Kyeongman Jeon, Chi Ryang Chung, Jeong Hoon Yang, Chi-Min Park, Gee Young Suh

**Affiliations:** 1 Department of Critical Care Medicine, Samsung Medical Center, Sungkyunkwan University School of Medicine, Seoul, Republic of Korea; 2 Division of Pulmonary and Critical Care Medicine, Department of Medicine, Samsung Medical Center, Sungkyunkwan University School of Medicine, Seoul, Republic of Korea; 3 Department of Medicine, Weill Cornell Medical College, New York, NY USA; 4 Division of Pulmonary and Critical Medicine, Brigham and Women’s Hospital, Harvard Medical School, Boston, MA USA

**Keywords:** Acute lung injury, Critical illness, Pulmonary surfactant-associated protein D, Respiratory distress syndrome

## Abstract

**Background:**

Acute respiratory distress syndrome (ARDS) is potentially underrecognized by clinicians. Early recognition and subsequent optimal treatment of patients with ARDS may be facilitated by usage of biomarkers. Surfactant protein D (SP-D), a marker of alveolar epithelial injury, has been proposed as a potentially useful biomarker for diagnosis of ARDS in a few studies. We tried to validate the performance of plasma SP-D levels for diagnosis of ARDS.

**Methods:**

We conducted a retrospective analysis using data from three (two in USA and one in Korea) prospective biobank cohorts involving 407 critically ill patients admitted to medical intensive care unit (ICU). A propensity score matched analysis (patients with versus without ARDS, matched 1:1) was carried out using significant variables from multiple logistic regression. The diagnostic accuracy of plasma SP-D as a diagnostic marker of ARDS was assessed by receiver operating characteristic curve analysis.

**Results:**

Out of the 407 subjects included in this study, 39 (10%) patients fulfilled ARDS criteria. Patients with ARDS had higher SP-D levels in plasma (*p* < 0.01) and higher hospital-mortality (*p* < 0.001) than those without ARDS. Thirty eight subjects with ARDS (cases) were successfully matched for propensity for ARDS with 38 subjects without ARDS (controls). Plasma levels of SP-D were higher in cases with ARDS compared to their matched controls without ARDS [median 20.8 ng/mL (interquartile range, 12.7–38.4) versus 7.9 (4.1–17.0); *p* = 0.001]. The area under the receiver operating characteristic curve for SP-D for the diagnosis of ARDS was 0.71 (95% confidence intervals, 0.60–0.83). A cut-off point of 12.7 ng/mL for SP-D yielded sensitivity of 74% and specificity of 63%.

**Conclusions:**

High levels of SP-D within 48 h after ICU admission might serve as a diagnostic marker for ARDS in patients hospitalized in medical ICU. Further prospective trials are required to validate the diagnostic role of SP-D in ARDS, and if its usefulness is greater in direct than in indirect ARDS, as well as across different strata of severity of ARDS.

## Background

A recent large study on the epidemiology of acute respiratory distress syndrome (ARDS) found that as many as 10% of patients admitted to the intensive care unit (ICU) fulfill the diagnostic criteria for this syndrome [1]. Even more importantly, clinical recognition and subsequent appropriate treatment of patients with ARDS may be suboptimal, ranging from 51% (in mild cases) to 79% (in severe cases) [1]. Such findings highlight the difficulties associated with the diagnosis of ARDS [2] and may emphasize the necessity for the identification of diagnostic biomarkers for this clinical entity.

Several biomarkers have been examined with regard to their discriminatory ability for the diagnosis of ARDS [3]. Surfactant protein D (SP-D) has been proposed as such a candidate [4, 5]. SP-D is one of the surfactant-associated proteins which are mainly synthesized in alveolar epithelial type II cells and non-ciliated bronchiolar epithelium [6, 7]. Apart from its role in surfactant homeostasis, SP-D seems to contribute to regulation of lung inflammation [7]. Lung inflammation and injury affect the synthesis and secretion of SP-D from lung epithelial cells into the systematic circulation [7]. The above characteristics of SP-D provided the rationale for its examination as a biomarker in human lung diseases [8], such as ARDS.

The potential usefulness of SP-D as a diagnostic biomarker of ARDS has been indeed tested in a few studies [6, 9–11]. Extensive validation of the diagnostic performance of biomarkers in various settings and centers is necessary to establish their clinical usefulness [12]. The importance of validation studies is currently fully recognized in the biomedical field and such studies are eagerly promoted [13, 14].

Having the above considerations into mind (namely, the previous promising findings regarding SP-D and the need for extensive validation of the performance of any biomarker), we endeavoured to examine whether plasma levels of SP-D within 48 h after medical ICU admission could help in the diagnosis of ARDS in critically ill patients in Asian and US cohorts.

## Methods

This was a retrospective study involving 407 critically ill patients admitted to medical ICU and included in one of the following three ongoing prospective cohorts; namely, Samsung Medical Center (SMC), Seoul, Korea; New York-Presbyterian Hospital/Weill Cornell Medical Center (NYPH/WCMC), New York, USA; Brigham and Women’s Hospital (BWH), Boston, USA. Patient enrollment in the above ongoing cohorts started in 12/2012 (SMC), 10/2014 (NYPH/WCMC) and 07/2008 (BWH).

Critically ill patients (>18 years) were enrolled in the cohorts within 24 h of their admission in medical ICU. Details on exclusion criteria have been previously provided [15, 16]. In brief, the following exclusion criteria were applied: cognitive impairment, inability to provide informed consent (or lack of an appropriate legal representative to do so), admission to the hospital purely to facilitate comfort care, unwillingness to receive blood transfusion, and a hemoglobin level of less than 7 g/dL (or 8 g/dL for the SMC cohort) at hospital admission [15, 16]. Comprehensive clinical information, such as demographics and past medical history, was collected for enrolled patients, along with laboratory values, severity of illness scoring, presence of sepsis, ARDS and other organ failures. Within 48 h after ICU admission, plasma was obtained.

As the enrollment had started before the release of Sepsis-3 definitions [17], for this study sepsis and septic shock were defined according to the American College of Chest Physicians/Society of Critical Care Medicine consensus conference and its revised version published in 2003 [18]. Disease severity was evaluated according to the Acute Physiology and Chronic Health Evaluation (APACHE) II score at the time of enrollment. The Berlin definition was used for the identification of patients with ARDS. At least two experienced physicians reviewed daily chest radiographs and blood gases and determined by consensus whether a given patient had ARDS. Attempts were made to detect and exclude patients with alternative causes of acute respiratory failure, such as cardiogenic pulmonary edema.

### Measurement of biomarkers

Whole blood was drawn from each patient into EDTA-coated blood collection tubes. The samples were stored at 4 °C and centrifuged within 4 h at 490×g for 10 min. Plasma was separated and kept frozen at -80 °C until further analysis. SP-D was assayed in duplicate using the Human SP-D Duoset ELISA (Enzyme-Linked Immunosorbent Assay) kit (DY1920, R&D systems). For the quantification of SP-D, plasma was diluted 25 times with 1× diluent supplied and a calibration curve was conducted. Procalcitonin was measured in serum using an enzyme-linked fluorescent assay (Brahms Diagnostica GmbH, Germany) and the lower reference limit was 0.05 ng/mL. Lactate was measured in serum using an enzymatic colorimetric method (Roche Hitachi, Japan).

### Statistical analysis

Continuous variables were presented as median [interquartile range (IQR)] and compared using the non-parametric Mann-Whitney *U* test. For SP-D levels, mean [± standard deviation (SD)] were presented along with median (IQR) values. Categorical variables were presented as percentages and compared using Fisher’s exact test or ×^2^-test. In an attempt to reduce the effect of confounders between the compared groups (patients with versus without ARDS) in this observational study, we used a propensity score matching technique. Propensity scores were estimated, without regard to outcomes, using multiple logistic regression analysis with the following covariates: age, sex, study site, diagnosis of pneumonia, presence of endotracheal intubation, and APACHE II score. The propensity score matched pairs developed without replacement (a 1:1 match) according to Greedy algorithm. As an additional attempt to reduce the effect of confounders, we repeated the multiple logistic regression analysis by including independent variables which were statistically significant between all patients in the compared groups (patients with versus without ARDS). A receiver operating characteristic (ROC) curve was plotted for SP-D and the diagnostic accuracy of the biomarker was assessed by calculating the area under the ROC curve (AUC). The optimal cutoff value was identified using the Liu method. All analyses were carried out using Stata SE 13.1 for Mac (StataCorp LP, College Station, TX). A two tailed *P* value of less than 0.05 was considered to be significant.

## Results

Out of the 407 patients included in this study, 39 (10%) individuals fulfilled diagnostic criteria for ARDS. Pneumonia was the most common predisposing factor, as it was present in 34 (87%) patients with ARDS.

In Table 1, characteristics of 39 patients with ARDS and 368 patients without ARDS are depicted. Patients with ARDS as opposed to those without ARDS had a higher APACHE II score at ICU admission [median 27.0 (IQR, 21.0–33.0) vs median 22.0 (IQR, 15.0–28.0); *p* < 0.001] and were more likely to be endotracheally intubated (90% vs 49%; p < 0.001). Four (10%) out of the 39 patients with ARDS were not endotracheally intubated at the time of blood draw; three of those patients were on non-invasive mechanical ventilation or high-flow nasal oxygen, while the remaining patient was on spontaneously breathing. With regard to laboratory values, patients with ARDS had higher procalcitonin [median 7.6 ng/mL (IQR, 2.6–33.2) vs median 0.9 ng/mL (IQR, 0.2–3.7); p < 0.001], lower albumin [median 2.5 g/dL (IQR, 2.1–2.9) vs median 2.9 g/dL (IQR, 2.4–3.5); *p* < 0.01], but not higher lactate [median 2.4 mEq/L (IQR, 1.5–3.5) vs median 2.0 mEq/L (IQR, 1.3–3.4); *p* = 0.24], than those without ARDS. SP-D levels in plasma were higher in patients with ARDS [median 20.8 ng/mL (IQR, 12.7–38.4); mean 26.9 ± 19.3 ng/mL] compared to those without ARDS [median 7.9 ng/mL (IQR, 4.0–17.0); mean 13.8 ± 17.2 ng/mL] (p < 0.01). Median SP-D level in plasma was 21.4 ng/mL [(IQR, 13.2–38.4); mean 27.3 ± 19.6 ng/mL] in the group of patients with direct ARDS and 16.3 ng/mL [(IQR, 9.7–38.3); mean 24.3 ± 18.4 ng/mL] in the group of patients with indirect ARDS. Finally, subjects with ARDS had poorer outcomes than those without ARDS in terms of length of ICU stay [median 8.0 days (IQR, 5.0–15.0) vs median 3.0 days (IQR, 2.0–6.0); *p* < 0.001], ICU-mortality (18% vs 8%; p < 0.01) and hospital-mortality (36% vs 14%; p < 0.001).

**Table 1:** Characteristics, laboratory values, and outcomes of patients with and without ARDS included in the study

	Patients with ARDS	Patients without ARDS	*P*-value
Number	39	368	
Age	57.0 (45.0–67.0)	62.0 (50.0–72.0)	0.07
Female	13 (33.3)	139 (37.8)	0.59
Study site			0.40
*Korea*	18 (46.2)	132 (35.9)	
*New York*	7 (18.0)	92 (25.0)	
*Boston*	14 (35.9)	144 (39.1)	
APACHE II^a^	27.0 (21.0–33.0)	22.0 (15.0–28.0)	< 0.001
Sepsis^b^	16 (41.0)	170 (46.2)	0.54
Septic shock	8 (20.5)	53 (14.4)	0.31
Pneumonia	34 (87.2)	101 (27.5)	< 0.001
Intubation	35 (89.7)	181 (49.2)	< 0.001
Procalcitonin, ng/mL^c^	7.6 (2.6–33.2)	0.9 (0.2–3.7)	< 0.001
Creatinine, mg/dL^a^	0.8 (0.6–1.6)	1.0 (0.7–1.5)	0.28
Bilirubin, mg/dL^a^	1.05 (0.6–3.0)	0.7 (0.5–1.1)	0.14
pH in arterial blood^a^	7.35 (7.28–7.45)	7.39 (7.29–7.46)	0.49
PaO_2_, mmHg^a^	72.9 (62.8–98.3)	87.7 (69.3–113.3)	0.15
PaCO_2_, mmHg^a^	36.9 (29.8–44.9)	32.4 (26.0–40.9)	0.07
Lactate, mEq/L	2.4 (1.5–3.5)	2.0 (1.3–3.4)	0.24
Albumin, g/dL	2.5 (2.1–2.9)	2.9 (2.4–3.5)	0.009
SP-D, ng/mL	20.8 (12.7–38.4)	7.9 (4.0–17.0)	< 0.01
Length of ICU stay	8.0 (5.0–15.0)	3.0 (2.0–6.0)	< 0.001
ICU-mortality	7 (17.9)	28 (7.6)	0.008
Hospital-mortality	14 (35.9)	50 (13.6)	< 0.001

*Abbreviations: ARDS* acute respiratory distress syndrome, *COPD* Chronic Obstructive Pulmonary Disease, *APACHE* Acute Physiology and Chronic Health Evaluation, *PaO*_*2*_ arterial oxygen tension, *PaCO2* arterial carbon dioxide tension, *SP-D* Surfactant protein D, *ICU* intensive care unit

Data are presented as median (interquartile range) or as number (%)

^a^At admission to medical ICU

^b^It does not include patients with septic shock

^c^Data are available for 91 patients (13 patients with and 78 patients without ARDS)

### Propensity score matching

Thirty eight patients with ARDS (cases) were successfully matched for propensity for ARDS with 38 patients without ARDS (controls). The one unmatched subject with ARDS (case) was a 20 years old male with a plasma level of SP-D equal to 37 ng/mL. The 330 unmatched subjects without ARDS (unmatched controls) had a median age of 62 years (IQR, 51–72) and a median plasma level of SP-D equal to 7.9 ng/mL (IQR, 4.0–16.7).

In Table 2, characteristics of 38 matched case and control subjects are depicted. There was no difference between cases and controls in terms of APACHE II score at ICU admission, presence of septic shock, albumin, lactate, and mortality. SP-D levels in plasma were higher in cases with ARDS [median 20.8 ng/mL (IQR, 12.7–38.4); mean 27.7 ± 19.5 ng/mL] compared to their matched controls without ARDS [median 7.9 ng/mL (IQR, 4.1–17.0); mean 14.8 ± 15.5 ng/mL] (*p* = 0.001). Median SP-D level in plasma was 20.8 ng/mL [(IQR, 13.2–38.4); mean 27.0 ± 19.9 ng/mL] in cases with direct ARDS and 16.3 ng/mL [(IQR, 9.7–38.3); mean 24.3 ± 18.4 ng/mL] in cases with indirect ARDS. Procalcitonin levels were higher in cases with ARDS [median 6.0 ng/mL (IQR, 2.1–36.4)] than in their matched controls without ARDS [median 0.8 ng/mL (IQR, 0.2–4.8)] (*p* = 0.03). There was no correlation between procalcitonin and SP-D levels in 19 patients who were included in the propensity score matched analysis and for whom data on both biomarkers were available (Pearson’s correlation = 0.0009, *p* = 0.99).

**Table 2:** Characteristics, laboratory values, and outcomes of cases (patients with ARDS) and matched controls (patients without ARDS) included in the propensity score matched analysis

	Cases (patients with ARDS)	Controls (patients without ARDS)	P-value
Number	38	38	
Age	57.0 (45.0–67.0)	62.0 (50.0–72.0)	0.53
Female	13 (34.2)	13 (34.2)	1.00
Study site			0.16
*Korea*	18 (46.2)	10 (25.6)	
*New York*	7 (17.9)	10 (25.6)	
*Boston*	14 (35.9)	19 (48.7)	
APACHE II^a^	27.5 (24.0–33.0)	26.0 (18.0–35.0)	0.62
Sepsis^b^	16 (42.1)	33 (86.8)	<0.001
Septic shock	7 (18.4)	4 (10.5)	0.33
Pneumonia	33 (86.8)	33 (86.8)	1.00
Intubation	34 (89.5)	32 (84.2)	0.55
Procalcitonin, ng/mL^c^	6.0 (2.1–36.4)	0.8 (0.2–4.8)	0.03
Creatinine, mg/dL^a^	0.8 (0.6–1.6)	1.0(0.7–1.9)	0.48
Bilirubin, mg/dL^a^	1.0 (0.6–3.0)	3.1 (0.5–6.1)	0.61
pH in arterial blood^a^	7.34 (7.28–7.45)	7.40 (7.28–7.46)	0.79
PaO_2_, mmHg^a^	72.9 (62.8–98.3)	87.7 (69.3–113.3)	0.92
PaCO_2_, mmHg^a^	36.9 (29.8–44.9)	32.4 (26.0–40.9)	0.82
Lactate, mEq/L	2.4 (1.5–3.5)	2.0 (1.3–3.4)	0.23
Albumin, g/dL	2.5 (2.1–3.0)	2.9 (2.4–3.5)	0.2
SP-D, ng/mL	20.8 (12.7–38.4)	7.9 (4.1–17.0)	0.001
Length of ICU stay	8.0 (5.0–15.0)	4.0 (2.0–10.0)	0.006
ICU Mortality	7 (18.4)	6 (15.8)	0.76
In-hospital Mortality	14 (35.9)	10 (26.3)	0.32

*Abbreviations: ARDS* acute respiratory distress syndrome, *APACHE* Acute Physiology and Chronic Health Evaluation, *PaO*_*2*_ arterial oxygen tension, *PaCO*_*2*_ arterial carbon dioxide tension, *SP-D* Surfactant protein D, *ICU* intensive care unit

Data are presented as median (interquartile range) or as number (%)

^a^At admission to medical ICU

^b^It does not include patients with septic shock

^c^Data are available for 19 patients (12 patients with and 7 patients without ARDS)

**Fig. 1:**
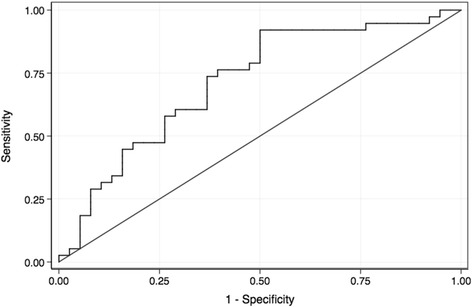
Receiver operating characteristic (ROC) curve of plasma Surfactant Protein D (SP-D) for discriminating patients with ARDS (*n* = 38) versus patients without ARDS (n = 38). The area under the ROC curve (AUC) for SP-D was 0.71 (95% confidence intervals, 0.60–0.83). A cut-off point of 12.7 ng/mL for SP-D yielded sensitivity of 74% and specificity of 63%

As depicted in Fig. 1, the AUC for SP-D for the diagnosis of ARDS was 0.71 (95% confidence intervals, 0.60–0.83). A cut-off point of 12.7 ng/mL for SP-D yielded sensitivity of 74% and specificity of 63%.

## Discussion

Using data from two US and one Asian cohort of critically ill patients admitted to the medical ICU, we found that plasma levels of SP-D within 48 h after ICU admission were significantly higher in patients with ARDS compared to patients without ARDS. We also found that plasma levels of SP-D provide sufficient discrimination for diagnosis of ARDS in medical ICU patients.

Our findings validate those reported by Ware et al.*,* who identified SP-D as an important component of a panel of biomarkers which provided good discrimination for diagnosis of ARDS in patients with severe sepsis [10]. Interestingly, the AUC for SP-D at the study enrollment day was exactly same (0.71) both in our study and the study of Ware et al. [10]. Our findings are also in line with those reported by Greene et al., who showed that serum SP-D levels were higher in 35 patients with ARDS than in 19 patients at risk for but without ARDS [6]. The study by Greene et al. however was single-center and focused on kinetics of surfactant proteins [6]. In a recently published sub-study to the Procalcitonin And Survival Study (PASS), Jensen et al. also reported that serum levels of SP-D were higher in patients with than without ARDS [19]. On the other hand, in a study by Determann et al.*,* plasma SP-D levels at the initiation of mechanical ventilation were double in 16 patients with ARDS than in 20 patients without ARDS (275 ng/ml and 140 ng/ml, *p* = 0.09); however, this difference did not reach statistical significance, because that study was presumably underpowered [9].

In our study, the great majority (87%) of patients with ARDS had pneumonia as predisposing factor; i.e. they presented with direct ARDS. ARDS is a heterogeneous syndrome, which can be divided according to the predisposing factor into direct (e.g. due to pneumonia) and indirect (e.g. due to pancreatitis or trauma). Calfee et al. reported that plasma SP-D levels (a marker of lung epithelial injury) were higher in patients with direct than in patients with indirect ARDS [11]. Plasma SP-D could be used as a biomarker for the identification of patients with direct ARDS who may benefit from therapies targeting specifically the lung epithelium.

Our study has the inherent limitations of a retrospectively observational study. However, we tried to reduce the effect of confounders by applying a widely accepted propensity score matching technique. Also, one may criticize the fact that we did not examine the performance of other biomarkers of lung epithelial injury, such as the receptor for advanced glycation end-products. We chose to focus on SP-D, because it has been previously reported that SP-D has better discriminatory ability than other epithelial injury biomarkers for diagnosis of ARDS [10]. Also, we did not examine whether SP-D could predict the outcome of patients with ARDS. Although some studies had shown that altered levels of plasma SP-D early in the course of ARDS are associated with worse clinical outcomes [7, 20], a more recent and larger study seemed to contradict those findings [11]. Thus, we chose to focus on the potential usefulness of SP-D as a diagnostic rather than a predictive biomarker for ARDS.

In addition, although SP-D may be better than other epithelial injury markers for diagnosis of ARDS [10], its AUC of 0.71 is not excellent [21]. Thus, it may be more useful as a component of a panel of biomarkers than as a single biomarker for diagnosis of ARDS [10]. Also, data on procalcitonin levels were available for 91 (22%) out of the 407 patients included in the study and we did not evaluate the strength of combining data for both procalcitonin and SP-D for the diagnosis of ARDS. Also, our sample size was not enough to allow for a stratification of the diagnostic power of SP-D according to the severity of ARDS. Finally, we understand that one may question the novelty and impact on the field of our validation study. However, we share the opinion that validation studies are indispensable in the effort to address irreproducibility in research [13, 14]. It is noteworthy that one of the largest ongoing randomized controlled trials in the field of ARDS, namely the Reevaluation of Systemic Early Neuromuscular Blockade (ROSE) trial (NCT02509078) carried out by the Clinical Trials Network for the Prevention and Early Treatment of Acute Lung Injury (PETAL) and funded by the National Heart, Lung, and Blood Institute (NHLBI), attempts to validate the results of a previously published trial [22].

## Conclusions

Our results validate those of previous studies showing that plasma SP-D has acceptable discriminatory ability for the diagnosis of ARDS. Such findings seem to provide sufficient support for further consideration of SP-D as a promising biomarker for diagnosis of ARDS. Further prospective trials are required to validate the diagnostic role of SP-D in ARDS, and if its usefulness is greater in direct than in indirect ARDS, as well as across different strata of severity of ARDS.

## Abbreviations

APACHE: Acute Physiology and Chronic Health Evaluation
ARDS: acute respiratory distress syndrome
AUC: area under the ROC curve
BWH: Brigham and Women’s Hospital
ICU: intensive care units
IQR: interquartile range
NYPH-WCMC: New York-Presbyterian Hospital/Weill Cornell Medical Center
ROC: receiver operating characteristic
SMC: Samsung Medical Center
SP-D: Surfactant protein D
